# (1*R*,2*S*)-[(*R*)-1-(2-Hy­droxy­naphthalen-1-yl)naphthalen-2-yl] 2-ethynyl­cyclo­propane-1-carboxyl­ate

**DOI:** 10.1107/S1600536811011627

**Published:** 2011-05-07

**Authors:** Jinlong Fan, Zhaohai Qin

**Affiliations:** a College of Science, China Agricultural University, 100094 Beijing, People’s Republic of China

## Abstract

In the crystal structure of the title compound, C_26_H_18_O_3_, mol­ecules with stereochemistry (1*R*,2S,*R*), are connected by O—H⋯O hydrogen bonds, forming chains.

## Related literature

The title structure is a stable cyclo­propane formate ester inter­mediate in the synthesis of abscisic acid analogues. (1*S*)-(+)-Abscisic acid is an important phytohormone with many functions in higher plants including roles in seed germination, development and dormancy, regulating the stomatal movements and improving stress tolerance, see: Frey *et al.* (1999 ▶); Jiang & Zhang (2004 ▶). For the synthesis of cyclo­propane formate ester, see: Reichelt & Martin (2006 ▶); Boche & Lohrenz (2001 ▶); Lebel *et al.* (2003 ▶); Molander & Etter (1987 ▶). 
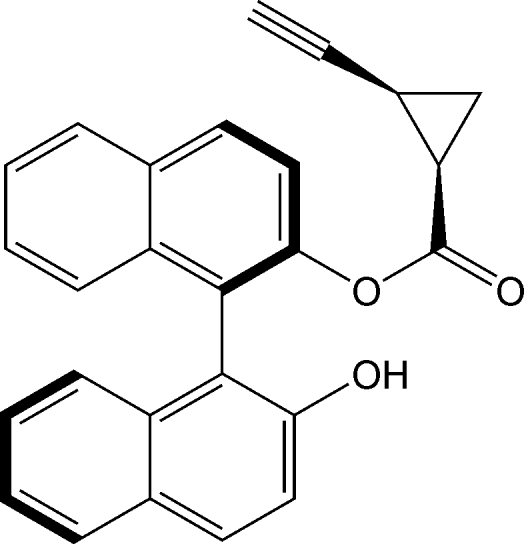


## Experimental

### 

#### Crystal data

C_26_H_18_O_3_
*M* *_r_* = 378.40
Orthorhombic, 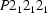

*a* = 8.0376 (11) Å
*b* = 12.0600 (17) Å
*c* = 20.324 (3) Å
*V* = 1970.1 (5) Å^3^
*Z* = 4
Cu *K*α radiation
μ = 0.66 mm^−1^
*T* = 173 K
0.65 × 0.48 × 0.35 mm

#### Data collection

Rigaku R-AXIS RAPID IP area-detector diffractometer
Absorption correction: multi-scan (*ABSCOR*; Higashi, 2001 ▶) *T*_min_ = 0.673, *T*_max_ = 0.801
13939 measured reflections
3554 independent reflections
3391 reflections with *I* > 2σ(*I*)
*R*_int_ = 0.032

#### Refinement

*R*[*F* ^2^ > 2σ(*F* ^2^)] = 0.032
*wR*(*F* ^2^) = 0.074
*S* = 1.07
3554 reflections
262 parameters
H-atom parameters constrained
Δρ_max_ = 0.11 e Å^−3^
Δρ_min_ = −0.13 e Å^−3^
Absolute structure: Flack (1983 ▶), 1471 Friedel pairs
Flack parameter: −0.06 (19)

### 

Data collection: *RAPID-AUTO* (Rigaku 1998 ▶); cell refinement: *RAPID-AUTO*; data reduction: *RAPID-AUTO*; program(s) used to solve structure: *SHELXS97* (Sheldrick, 2008 ▶); program(s) used to refine structure: *SHELXL97* (Sheldrick, 2008 ▶); molecular graphics: *XP* in *SHELXTL* (Sheldrick, 2008 ▶); software used to prepare material for publication: *SHELXL97*.

## Supplementary Material

Crystal structure: contains datablocks I, global. DOI: 10.1107/S1600536811011627/gw2095sup1.cif

Structure factors: contains datablocks I. DOI: 10.1107/S1600536811011627/gw2095Isup2.hkl

Additional supplementary materials:  crystallographic information; 3D view; checkCIF report

## Figures and Tables

**Table 1:** Hydrogen-bond geometry (Å, °)

*D*—H⋯*A*	*D*—H	H⋯*A*	*D*⋯*A*	*D*—H⋯*A*
O1—H1*A*⋯O3^i^	0.84	2.01	2.8520 (16)	177

Symmetry code: (i) 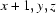.

**Fig. 1.**
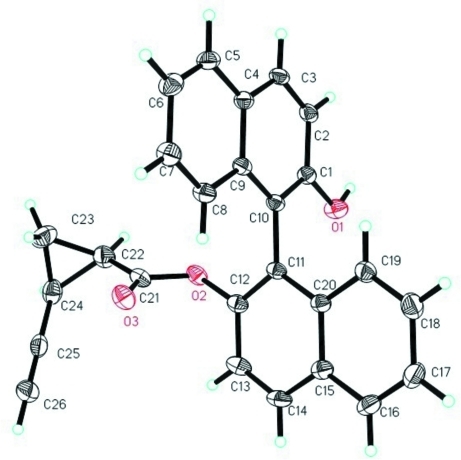
Ellipsoid plot.

**Fig. 2.**
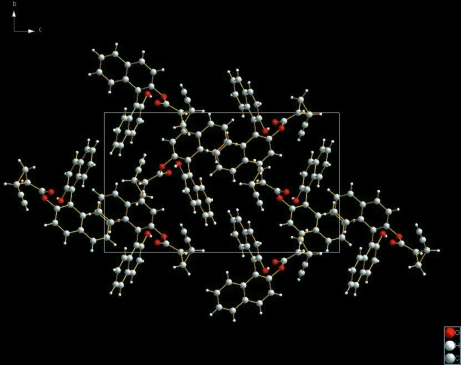
Packing diagram.

## supplementary crystallographic information

### Comment

(1*S*)-(+)-Abscisic acid (ABA) is an important phytohormone with many functions in higher plants including roles in seed germination, development and dormancy, regulating the stomatal movements and improving stress tolerance (Frey *et al.*, 1999; Jiang *et al.*, 2004). The title structure, C_26_H_18_O_3_, is a stable cyclopropane formate ester intermediate in the synthesis of Abscisic acid analoge. During the course of our study, we remove the protected group, trimethanesilicon, from the compound of (1*R*,2S)-((*R*)-2'-hydroxy-1,1'-binaphthyl-2-yl) 2-((trimethylsilyl)ethynyl)cyclopropanecarboxylate, to obtain the title compoud. In this paper, we reported crystal structure of the title compound.

The crystal structure is shown in Figure1. The crystal structure consists of one three-membered rings(A) and two naphthalene nucleus(B/C). The C22 is *R* configuration with the dihedral angles C22—C23—C24—C25 = 109.07 (51)°. The C24 is S configuration with the dihedral angles C21—C22—C24—C23 = 108.01 (53)°. The two naphthalene nucleus(B/C) is *R* configuration with the dihedral angles C(1)—C(10)—C(11)—C(20) = 92.85 (07)°. the compounds are connected by O1—H1A···O3i,hydrogen bonds (2.852, Symmetry code: (i) *x* + 1, *y*, *z*.).

### Experimental

A solution of (1*R*,2S)-((*R*)-2'-hydroxy-1,1'-binaphthyl-2-yl) 2-((trimethylsilyl)ethynyl)cyclopropanecarboxylate (360 mg, 0.775 mmol) was cooled to 0 ^°^C, and TBAF (1.0 *M* THF solution, 1.16 ml, 1.16 mmol) was added. The resulting solution was stirred for 3 h at 0^°^C. Saturated NH4Cl solution was added, and the aqueous phase was extracted with Et_2_O. The organic phase was washed with brine, dried over Na_2_SO_4_, filtered, and concentrated. The residue was purified by column chromatography on silica gel (1:1 hexane/benzene) to provide 23*a* (229.4 mg, 78.3%). 1H NMR (500 MHz, CDCl_3_,TMS): δ 0.9566–1.1.0282 (m,1H), 1.0925 - 1.1524(m, 1H), 1.6355–1.7887 (m, 2 H), 1.9454–1.9527 (d, 1 H), 5.3893 (s, 1 H), 7.0617–7.0659(q,1 H), 7.2167–7.3231 (m, 6H), 7.4202–7.4686(q, 2 H), 7.8104–7.9397 (ddd,3H), 8.0015–8.0309(d, 1 H); 13 C NMR (75 MHz, CDCl_3_): δ10.027, 14.828, 20.324, 29.639, 68.373, 80.191, 114.146, 118.643, 121.931, 123.372, 124.595, 125.744, 126.186, 126.542, 127.313, 127.910, 128.984, 130.116, 130.545, 132.182, 133.450, 133.577. The compound was redissolved in n-hexane (20 ml) and dichloromethane (5 ml), and crystals suitable for X-ray analysis were grown from slow evaporation of the solvent at room temperature.

### Refinement

H atoms on C were placed in idealized positions with C—H distances 0.95 - 0.99 Å and thereafter treated as riding. *U*_iso_ for H were assigned as 1.2 times *U*_eq_ of the attached C atom. The result of refinement is *R*[*F*^2^ > 2σ(*F*^2^)] =0.032, *wR*(*F*^2^) = 0.074, Flack parameter: -0.06 (19), so the absolute configuration can be determined.

### Crystal data

**Table:**

C_26_H_18_O_3_	*F*(000) = 792
*M**_r_* = 378.40	*D*_x_ = 1.276 Mg m^−^^3^
Orthorhombic, *P*2_1_2_1_2_1_	Cu *K*α radiation, λ = 1.54186 Å
Hall symbol: P 2ac 2ab	Cell parameters from 14013 reflections
*a* = 8.0376 (11) Å	θ = 3.1–68.2°
*b* = 12.0600 (17) Å	µ = 0.66 mm^−^^1^
*c* = 20.324 (3) Å	*T* = 173 K
*V* = 1970.1 (5) Å^3^	Block, colorless
*Z* = 4	0.65 × 0.48 × 0.35 mm

### Data collection

**Table:**

Rigaku R-AXIS RAPID IP area-detector diffractometer	3554 independent reflections
Radiation source: rotating anode	3391 reflections with *I* > 2σ(*I*)
graphite	*R*_int_ = 0.032
ω scans	θ_max_ = 68.2°, θ_min_ = 4.3°
Absorption correction: multi-scan (*ABSCOR*; Higashi, 2001)	*h* = −9→9
*T*_min_ = 0.673, *T*_max_ = 0.801	*k* = −12→14
13939 measured reflections	*l* = −24→24

### Refinement

**Table:**

Refinement on *F*^2^	Secondary atom site location: difference Fourier map
Least-squares matrix: full	Hydrogen site location: inferred from neighbouring sites
*R*[*F*^2^ > 2σ(*F*^2^)] = 0.032	H-atom parameters constrained
*wR*(*F*^2^) = 0.074	*w* = 1/[σ^2^(*F*_o_^2^) + (0.0274*P*)^2^ + 0.3717*P*] where *P* = (*F*_o_^2^ + 2*F*_c_^2^)/3
*S* = 1.07	(Δ/σ)_max_ = 0.001
3554 reflections	Δρ_max_ = 0.11 e Å^−^^3^
262 parameters	Δρ_min_ = −0.13 e Å^−^^3^
0 restraints	Absolute structure: Flack (1983), 1471 Friedel pairs
Primary atom site location: structure-invariant direct methods	Flack parameter: −0.06 (19)

### Special details

**Table:**

Geometry. All e.s.d.'s (except the e.s.d. in the dihedral angle between two l.s. planes) are estimated using the full covariance matrix. The cell e.s.d.'s are taken into account individually in the estimation of e.s.d.'s in distances, angles and torsion angles; correlations between e.s.d.'s in cell parameters are only used when they are defined by crystal symmetry. An approximate (isotropic) treatment of cell e.s.d.'s is used for estimating e.s.d.'s involving l.s. planes.
Refinement. Refinement of *F*^2^ against ALL reflections. The weighted *R*-factor *wR* and goodness of fit *S* are based on *F*^2^, conventional *R*-factors *R* are based on *F*, with *F* set to zero for negative *F*^2^. The threshold expression of *F*^2^ > σ(*F*^2^) is used only for calculating *R*-factors(gt) *etc*. and is not relevant to the choice of reflections for refinement. *R*-factors based on *F*^2^ are statistically about twice as large as those based on *F*, and *R*- factors based on ALL data will be even larger.

### Fractional atomic coordinates and isotropic or equivalent isotropic displacement parameters (Å^2^)

**Table:**

	*x*	*y*	*z*	*U*_iso_*/*U*_eq_	
O1	0.82773 (14)	0.36744 (9)	0.81560 (6)	0.0412 (3)	
H1A	0.9241	0.3840	0.8030	0.062*	
O2	0.41585 (13)	0.38707 (9)	0.74612 (5)	0.0360 (3)	
O3	0.15312 (14)	0.43096 (11)	0.77423 (6)	0.0443 (3)	
C1	0.75354 (19)	0.45851 (13)	0.84229 (7)	0.0294 (3)	
C2	0.84313 (19)	0.55862 (13)	0.85081 (8)	0.0326 (3)	
H2A	0.9556	0.5632	0.8367	0.039*	
C3	0.7695 (2)	0.64824 (13)	0.87909 (7)	0.0329 (4)	
H3A	0.8313	0.7147	0.8847	0.039*	
C4	0.6014 (2)	0.64350 (13)	0.90027 (7)	0.0306 (3)	
C5	0.5215 (2)	0.73583 (14)	0.92935 (8)	0.0374 (4)	
H5A	0.5808	0.8035	0.9340	0.045*	
C6	0.3614 (2)	0.72960 (14)	0.95074 (8)	0.0415 (4)	
H6A	0.3103	0.7920	0.9709	0.050*	
C7	0.2721 (2)	0.63017 (15)	0.94282 (8)	0.0402 (4)	
H7A	0.1599	0.6260	0.9573	0.048*	
C8	0.3447 (2)	0.53947 (14)	0.91456 (8)	0.0350 (4)	
H8A	0.2821	0.4732	0.9097	0.042*	
C9	0.51197 (19)	0.54257 (12)	0.89239 (7)	0.0280 (3)	
C10	0.59105 (18)	0.44935 (12)	0.86295 (7)	0.0270 (3)	
C11	0.50195 (18)	0.34074 (12)	0.85607 (7)	0.0279 (3)	
C12	0.4190 (2)	0.31262 (13)	0.79970 (7)	0.0319 (3)	
C13	0.3400 (2)	0.21003 (15)	0.79066 (8)	0.0419 (4)	
H13A	0.2861	0.1937	0.7502	0.050*	
C14	0.3408 (2)	0.13429 (14)	0.83998 (9)	0.0406 (4)	
H14A	0.2880	0.0646	0.8338	0.049*	
C15	0.4194 (2)	0.15809 (13)	0.90047 (8)	0.0341 (4)	
C16	0.4193 (2)	0.08122 (14)	0.95328 (9)	0.0411 (4)	
H16A	0.3670	0.0112	0.9477	0.049*	
C17	0.4927 (2)	0.10604 (15)	1.01160 (10)	0.0460 (4)	
H17A	0.4917	0.0534	1.0463	0.055*	
C18	0.5704 (2)	0.20966 (16)	1.02082 (9)	0.0460 (4)	
H18A	0.6197	0.2271	1.0620	0.055*	
C19	0.5753 (2)	0.28568 (15)	0.97044 (8)	0.0363 (4)	
H19A	0.6297	0.3548	0.9770	0.044*	
C20	0.50037 (19)	0.26228 (13)	0.90897 (7)	0.0296 (3)	
C21	0.2672 (2)	0.43827 (14)	0.73624 (8)	0.0355 (4)	
C22	0.2674 (2)	0.49921 (16)	0.67393 (9)	0.0469 (4)	
H22A	0.3780	0.5094	0.6522	0.056*	
C23	0.1432 (3)	0.59106 (16)	0.66459 (12)	0.0627 (6)	
H23A	0.0669	0.6075	0.7016	0.075*	
H23B	0.1790	0.6568	0.6391	0.075*	
C24	0.1168 (2)	0.48554 (16)	0.62786 (9)	0.0482 (5)	
H24A	0.1427	0.4888	0.5798	0.058*	
C25	−0.0173 (2)	0.41205 (15)	0.64584 (9)	0.0424 (4)	
C26	−0.1269 (3)	0.35242 (17)	0.66089 (10)	0.0527 (5)	
H26	−0.2153	0.3043	0.6730	0.063*	

### Atomic displacement parameters (Å^2^)

**Table:**

	*U*^11^	*U*^22^	*U*^33^	*U*^12^	*U*^13^	*U*^23^
O1	0.0302 (6)	0.0372 (6)	0.0561 (7)	−0.0033 (5)	0.0157 (5)	−0.0033 (6)
O2	0.0278 (6)	0.0474 (7)	0.0327 (5)	−0.0075 (5)	0.0028 (5)	0.0014 (5)
O3	0.0312 (6)	0.0593 (8)	0.0424 (6)	−0.0025 (6)	0.0080 (5)	0.0005 (6)
C1	0.0271 (8)	0.0314 (8)	0.0295 (7)	−0.0030 (6)	0.0028 (6)	0.0018 (6)
C2	0.0255 (8)	0.0375 (9)	0.0348 (8)	−0.0076 (7)	−0.0016 (6)	0.0075 (7)
C3	0.0351 (9)	0.0309 (8)	0.0326 (8)	−0.0127 (7)	−0.0058 (7)	0.0051 (7)
C4	0.0351 (9)	0.0284 (8)	0.0283 (7)	−0.0050 (7)	−0.0044 (6)	0.0023 (6)
C5	0.0478 (11)	0.0284 (8)	0.0360 (8)	−0.0054 (7)	−0.0053 (7)	−0.0035 (7)
C6	0.0493 (11)	0.0341 (9)	0.0410 (9)	0.0048 (8)	0.0017 (8)	−0.0082 (8)
C7	0.0348 (9)	0.0434 (10)	0.0426 (9)	0.0005 (8)	0.0057 (7)	−0.0074 (8)
C8	0.0319 (8)	0.0348 (9)	0.0384 (8)	−0.0041 (7)	0.0026 (7)	−0.0075 (7)
C9	0.0282 (7)	0.0293 (8)	0.0265 (7)	−0.0046 (6)	−0.0021 (6)	0.0001 (6)
C10	0.0246 (7)	0.0278 (8)	0.0284 (7)	−0.0051 (6)	−0.0003 (6)	0.0008 (6)
C11	0.0212 (7)	0.0294 (8)	0.0330 (8)	−0.0031 (6)	0.0055 (6)	−0.0057 (6)
C12	0.0268 (8)	0.0353 (8)	0.0336 (8)	−0.0046 (7)	0.0062 (6)	−0.0031 (7)
C13	0.0388 (10)	0.0458 (10)	0.0411 (9)	−0.0132 (8)	0.0013 (7)	−0.0116 (8)
C14	0.0368 (9)	0.0315 (9)	0.0536 (10)	−0.0108 (7)	0.0050 (8)	−0.0121 (8)
C15	0.0264 (8)	0.0299 (8)	0.0458 (9)	0.0004 (7)	0.0098 (7)	−0.0029 (7)
C16	0.0326 (9)	0.0274 (8)	0.0633 (11)	0.0020 (7)	0.0120 (8)	0.0060 (8)
C17	0.0343 (9)	0.0432 (10)	0.0604 (11)	0.0055 (8)	0.0063 (9)	0.0200 (9)
C18	0.0352 (9)	0.0567 (11)	0.0461 (10)	−0.0030 (9)	−0.0038 (8)	0.0114 (9)
C19	0.0289 (8)	0.0394 (9)	0.0405 (9)	−0.0038 (7)	−0.0004 (7)	0.0037 (7)
C20	0.0219 (8)	0.0285 (8)	0.0385 (8)	−0.0010 (6)	0.0056 (6)	−0.0024 (6)
C21	0.0280 (8)	0.0412 (10)	0.0374 (8)	−0.0076 (7)	0.0006 (7)	−0.0033 (7)
C22	0.0373 (10)	0.0528 (11)	0.0505 (10)	−0.0067 (8)	0.0051 (8)	0.0117 (9)
C23	0.0663 (14)	0.0381 (10)	0.0838 (15)	−0.0042 (10)	0.0040 (12)	0.0158 (11)
C24	0.0474 (11)	0.0546 (12)	0.0426 (9)	0.0085 (9)	0.0007 (8)	0.0120 (9)
C25	0.0414 (10)	0.0404 (10)	0.0452 (10)	0.0136 (8)	−0.0111 (8)	−0.0059 (8)
C26	0.0463 (12)	0.0469 (11)	0.0649 (12)	−0.0016 (9)	−0.0109 (9)	−0.0083 (10)

### Geometric parameters (Å, °)

**Table:**

O1—C1	1.3623 (18)	C13—C14	1.356 (2)
O1—H1A	0.8400	C13—H13A	0.9500
O2—C21	1.3596 (19)	C14—C15	1.412 (2)
O2—C12	1.4116 (18)	C14—H14A	0.9500
O3—C21	1.2021 (19)	C15—C16	1.418 (2)
C1—C10	1.376 (2)	C15—C20	1.426 (2)
C1—C2	1.416 (2)	C16—C17	1.357 (3)
C2—C3	1.360 (2)	C16—H16A	0.9500
C2—H2A	0.9500	C17—C18	1.410 (3)
C3—C4	1.419 (2)	C17—H17A	0.9500
C3—H3A	0.9500	C18—C19	1.375 (2)
C4—C5	1.415 (2)	C18—H18A	0.9500
C4—C9	1.423 (2)	C19—C20	1.415 (2)
C5—C6	1.360 (3)	C19—H19A	0.9500
C5—H5A	0.9500	C21—C22	1.464 (2)
C6—C7	1.407 (2)	C22—C23	1.503 (3)
C6—H6A	0.9500	C22—C24	1.539 (3)
C7—C8	1.366 (2)	C22—H22A	1.0000
C7—H7A	0.9500	C23—C24	1.491 (3)
C8—C9	1.418 (2)	C23—H23A	0.9900
C8—H8A	0.9500	C23—H23B	0.9900
C9—C10	1.423 (2)	C24—C25	1.442 (3)
C10—C11	1.4994 (19)	C24—H24A	1.0000
C11—C12	1.368 (2)	C25—C26	1.178 (3)
C11—C20	1.432 (2)	C26—H26	0.9500
C12—C13	1.403 (2)		
			
C1—O1—H1A	109.5	C15—C14—H14A	119.7
C21—O2—C12	114.75 (12)	C14—C15—C16	121.74 (15)
O1—C1—C10	118.17 (13)	C14—C15—C20	119.28 (15)
O1—C1—C2	120.89 (13)	C16—C15—C20	118.98 (15)
C10—C1—C2	120.90 (14)	C17—C16—C15	121.09 (16)
C3—C2—C1	120.53 (14)	C17—C16—H16A	119.5
C3—C2—H2A	119.7	C15—C16—H16A	119.5
C1—C2—H2A	119.7	C16—C17—C18	120.29 (17)
C2—C3—C4	120.70 (14)	C16—C17—H17A	119.9
C2—C3—H3A	119.6	C18—C17—H17A	119.9
C4—C3—H3A	119.6	C19—C18—C17	120.31 (17)
C5—C4—C3	121.82 (15)	C19—C18—H18A	119.8
C5—C4—C9	119.42 (14)	C17—C18—H18A	119.8
C3—C4—C9	118.77 (14)	C18—C19—C20	120.81 (15)
C6—C5—C4	121.29 (15)	C18—C19—H19A	119.6
C6—C5—H5A	119.4	C20—C19—H19A	119.6
C4—C5—H5A	119.4	C19—C20—C15	118.50 (14)
C5—C6—C7	119.55 (16)	C19—C20—C11	121.81 (13)
C5—C6—H6A	120.2	C15—C20—C11	119.69 (14)
C7—C6—H6A	120.2	O3—C21—O2	122.84 (15)
C8—C7—C6	120.84 (16)	O3—C21—C22	126.36 (16)
C8—C7—H7A	119.6	O2—C21—C22	110.79 (14)
C6—C7—H7A	119.6	C21—C22—C23	118.58 (17)
C7—C8—C9	121.15 (15)	C21—C22—C24	118.15 (15)
C7—C8—H8A	119.4	C23—C22—C24	58.66 (13)
C9—C8—H8A	119.4	C21—C22—H22A	116.4
C8—C9—C4	117.75 (14)	C23—C22—H22A	116.4
C8—C9—C10	122.41 (14)	C24—C22—H22A	116.4
C4—C9—C10	119.84 (13)	C24—C23—C22	61.87 (13)
C1—C10—C9	119.25 (13)	C24—C23—H23A	117.6
C1—C10—C11	119.66 (13)	C22—C23—H23A	117.6
C9—C10—C11	121.06 (12)	C24—C23—H23B	117.6
C12—C11—C20	117.42 (13)	C22—C23—H23B	117.6
C12—C11—C10	121.83 (13)	H23A—C23—H23B	114.7
C20—C11—C10	120.75 (13)	C25—C24—C23	120.26 (17)
C11—C12—C13	123.30 (15)	C25—C24—C22	119.94 (15)
C11—C12—O2	119.81 (13)	C23—C24—C22	59.47 (13)
C13—C12—O2	116.85 (14)	C25—C24—H24A	115.3
C14—C13—C12	119.67 (15)	C23—C24—H24A	115.3
C14—C13—H13A	120.2	C22—C24—H24A	115.3
C12—C13—H13A	120.2	C26—C25—C24	179.6 (2)
C13—C14—C15	120.59 (15)	C25—C26—H26	180.0
C13—C14—H14A	119.7		
			
O1—C1—C2—C3	−178.23 (13)	C11—C12—C13—C14	1.5 (3)
C10—C1—C2—C3	−0.6 (2)	O2—C12—C13—C14	179.06 (15)
C1—C2—C3—C4	−0.4 (2)	C12—C13—C14—C15	0.5 (3)
C2—C3—C4—C5	−179.49 (15)	C13—C14—C15—C16	178.77 (16)
C2—C3—C4—C9	1.1 (2)	C13—C14—C15—C20	−0.9 (2)
C3—C4—C5—C6	−178.58 (15)	C14—C15—C16—C17	−178.77 (16)
C9—C4—C5—C6	0.9 (2)	C20—C15—C16—C17	0.9 (2)
C4—C5—C6—C7	−1.1 (2)	C15—C16—C17—C18	0.2 (3)
C5—C6—C7—C8	0.7 (3)	C16—C17—C18—C19	−1.2 (3)
C6—C7—C8—C9	0.0 (3)	C17—C18—C19—C20	1.1 (2)
C7—C8—C9—C4	−0.3 (2)	C18—C19—C20—C15	0.1 (2)
C7—C8—C9—C10	179.83 (15)	C18—C19—C20—C11	179.15 (15)
C5—C4—C9—C8	−0.1 (2)	C14—C15—C20—C19	178.64 (15)
C3—C4—C9—C8	179.33 (14)	C16—C15—C20—C19	−1.1 (2)
C5—C4—C9—C10	179.75 (13)	C14—C15—C20—C11	−0.4 (2)
C3—C4—C9—C10	−0.8 (2)	C16—C15—C20—C11	179.84 (14)
O1—C1—C10—C9	178.55 (13)	C12—C11—C20—C19	−176.82 (15)
C2—C1—C10—C9	0.9 (2)	C10—C11—C20—C19	3.0 (2)
O1—C1—C10—C11	0.4 (2)	C12—C11—C20—C15	2.2 (2)
C2—C1—C10—C11	−177.20 (14)	C10—C11—C20—C15	−177.97 (13)
C8—C9—C10—C1	179.69 (14)	C12—O2—C21—O3	8.2 (2)
C4—C9—C10—C1	−0.2 (2)	C12—O2—C21—C22	−170.69 (13)
C8—C9—C10—C11	−2.2 (2)	O3—C21—C22—C23	22.1 (3)
C4—C9—C10—C11	177.90 (13)	O2—C21—C22—C23	−159.11 (16)
C1—C10—C11—C12	−87.34 (18)	O3—C21—C22—C24	−45.6 (3)
C9—C10—C11—C12	94.59 (18)	O2—C21—C22—C24	133.24 (16)
C1—C10—C11—C20	92.87 (17)	C21—C22—C23—C24	−107.30 (19)
C9—C10—C11—C20	−85.20 (17)	C22—C23—C24—C25	109.08 (18)
C20—C11—C12—C13	−2.8 (2)	C21—C22—C24—C25	−1.6 (3)
C10—C11—C12—C13	177.41 (15)	C23—C22—C24—C25	−109.6 (2)
C20—C11—C12—O2	179.69 (12)	C21—C22—C24—C23	108.0 (2)
C10—C11—C12—O2	−0.1 (2)	C23—C24—C25—C26	−13 (38)
C21—O2—C12—C11	−107.04 (16)	C22—C24—C25—C26	57 (38)
C21—O2—C12—C13	75.28 (18)		

### Hydrogen-bond geometry (Å, °)

**Table:**

*D*—H···*A*	*D*—H	H···*A*	*D*···*A*	*D*—H···*A*
O1—H1A···O3^i^	0.84	2.01	2.8520 (16)	177

Symmetry codes: (i) *x*+1, *y*, *z*.
